# Divergent Egg‐Rejection Strategies Between Laying and Incubation Periods in the Green‐Backed Tit (*Parus monticolus*)

**DOI:** 10.1002/ece3.73404

**Published:** 2026-04-01

**Authors:** Ping Ye, Canchao Yang

**Affiliations:** ^1^ Key Laboratory of Southwest China Wildlife Resources Conservation (Ministry of Education) China West Normal University Nanchong China; ^2^ Ministry of Education Key Laboratory for Ecology of Tropical Islands, College of Life Sciences Hainan Normal University Haikou China

**Keywords:** avian brood parasitism, breeding stage, breeding time, cavity‐nesting host, egg recognition

## Abstract

Egg recognition and rejection constitute essential host defenses against brood parasitism, with rejection decisions reflecting evolutionary adaptations shaped by cost–benefit trade‐offs. Although long‐term studies have established correlations between egg rejection behavior and parasitism risk, it remains unclear whether hosts can dynamically adjust their defenses in response to stage‐specific parasitic threats across different breeding periods. We investigated temporal variation in egg recognition and rejection latency in the green‐backed tit ( *Parus monticolus* ), analyzing both seasonal patterns and stage‐specific responses within breeding cycles. Our findings reveal that while rejection behavior remained consistently precise throughout the breeding season without seasonal variation, we observed a striking stage‐dependent pattern: complete acceptance of parasitic eggs during laying followed by near‐total rejection during incubation. This biphasic response contrasts sharply with patterns reported in most other host species. We propose this unique recognition strategy stems from the tit's egg‐covering behavior during laying—an adaptation likely driven by nest predation pressure that temporarily suppresses anti‐parasitic defenses. These results demonstrate that green‐backed tits have evolved independent adaptive responses to distinct selective pressures: nest predation during egg‐laying and brood parasitism during incubation. This study provides new insights into the plasticity of host defenses and suggests that species with egg‐covering behaviors may develop specialized anti‐parasitic strategies. Our findings offer important implications for understanding the evolutionary dynamics of host–parasite interactions and establish a framework for investigating recognition behaviors in other cavity‐nesting hosts.

## Graphical abstract

Our results demonstrate that green‐backed tits have evolved independent adaptive responses to distinct selective pressures: nest predation during egg‐laying and brood parasitism during incubation. This study provides new insights into the plasticity of host defenses and suggests that species with egg‐covering behaviors may develop specialized anti‐parasitic strategies.

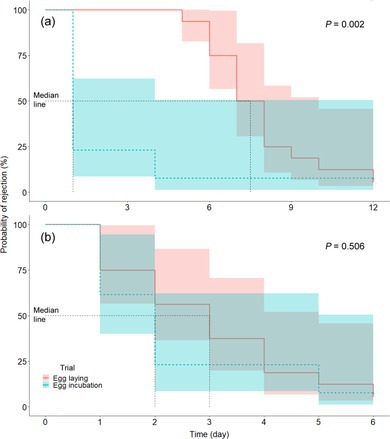

## 1 Introduction

Avian brood parasitism represents a remarkable evolutionary strategy in which parasitic species such as cuckoos (*Cuculus* spp.) lay their eggs in host nests to exploit parental care (Davies 2000). This parasitic relationship imposes substantial fitness costs on hosts, driving the evolution of sophisticated anti‐parasitic defenses including nest protection, egg recognition, and nestling discrimination (Davies 2011; Soler 2014). Among these, egg recognition and rejection constitute the most widespread and effective defense mechanisms, enabling hosts to identify and eliminate parasitic eggs (Davies and Brooke 1989).

However, maintaining effective egg recognition involves significant trade‐offs. Hosts must balance the benefits of rejecting parasite eggs against potential costs, including: (1) recognition errors leading to accidental rejection of their own eggs (Stokke et al. 2016; Liu, Wang, and Liang 2023), and (2) physical damage to host eggs during rejection attempts (Moskát 2005; Liu, Wang, and Liang 2023). Thus, optimal defense investment should be plastic and adjust dynamically to the level of parasitism threat. Indeed, the risk of parasitism is not static but varies over time, both across seasons and within a single breeding attempt (Brooke et al. 1998; Soler et al. 2020). For instance, in systems with summer‐migratory cuckoo parasites, hosts typically begin reproduction before the parasites arrive (Payne 2005), creating temporal variation in parasitism risk that depends on host laying schedules (Zhang et al. 2021). Additionally, most parasitism events occur during the host's egg‐laying stage (Lotem et al. 1995), suggesting that parasitism risk declines substantially during incubation. These temporal dynamics should theoretically favor plastic host defenses that adjust according to current parasitism risk.

However, empirical studies present conflicting patterns regarding temporal variation in egg rejection behavior. Some systems demonstrate clear stage‐ or season‐dependent rejection patterns. For example: Great reed warblers ( *Acrocephalus arundinaceus* ) reduced rejection rates from 75% to 25% over 12 years as cuckoo parasitism declined (Brooke et al. 1998). Red‐whiskered bulbuls ( *Pycnonotus jocosus* ) showed higher rejection rates during egg‐laying than incubation (Liu et al. 2021). Daurian redstarts ( *Phoenicurus auroreus* ) increased rejection rates seasonally in response to cuckoo arrival patterns (Zhang et al. 2021). Conversely, other studies report consistent rejection across stages: Warbling vireos ( *Vireo gilvus* ) showed no stage‐dependent rejection (Underwood and Sealy 2006). Oriental reed warblers ( *A. orientalis* ) maintained strong recognition even into the nestling stage (Ma et al. 2024). Five songbird species showed similar rejection rates across laying/incubation stages (Davies and Brooke 1989). Notably, most studies have focused on hosts that are frequently parasitized. Much less is known about species with strong defenses and little or no recorded parasitism. For these “enigmatic” hosts, a critical question is whether their egg recognition exhibits temporal plasticity and what drives it. In addition, most previous research has focused exclusively on rejection rates, while other critical aspects of recognition behavior—particularly rejection latency—remain understudied. The timing of rejection represents a crucial component of host defense strategies, as earlier parasite egg removal reduces incubation costs (Visser and Lessells 2001) and may be more sensitive to parasitism risk than simple rejection rates (Davies and Brooke 1989; Grim et al. 2011).

The green‐backed tit ( *Parus monticolus* ) is a cavity‐nesting species with a strong egg recognition capacity (Yang et al. 2019). However, it is not a commonly recorded host of the cuckoo. This dissociation between strong defense capability and the sheer absence of parasitism makes it an ideal system for studying whether their egg recognition exhibits temporal plasticity. Therefore, we employed a dual experimental approach, artificially parasitized nests during egg‐laying and monitored responses through incubation and conducted parallel experiments during incubation only. This design allowed us to examine stage‐specific variation in rejection behavior, individual response plasticity across breeding stages, and seasonal patterns in rejection rates and latency.

## 2 Materials and Methods

### 2.1 Study Area and Species

This study was conducted in Kuankuoshui National Nature Reserve, Guizhou Province, southwestern China (107°02′–107°14′ E, 28°06′–28°19′ N) from March to July of 2019–2021. The total area of the reserve is 26,231 ha, with altitudes ranging between 650 and 1762 m. The annual average temperature ranges from 11.7°C to 15.2°C while the annual average relative humidity is greater than 82% and the annual rainfall ranges between 1300 and 1350 mm (Yang et al. 2023; Ye et al. 2022). Our study area was situated in a subtropical moist mixed broadleaved forest at an altitude of approximately 1500 m, where more than 200 nest boxes (30 cm in depth with an entrance hole diameter of 4.5 cm) were set up to provide nesting sites for green‐backed tits ( *Parus monticolus* ).

The green‐backed tit is a small secondary cavity‐nesting bird widely distributed across central, southwestern regions, southern Tibet, and Taiwan of China. It lays a typical clutch size of approximately seven eggs with white backgrounds and reddish‐brown spots, and the egg incubation was executed solely by the female (Ye et al. 2021). A notable behavior of this species is that during the laying stage, the female covers the eggs with nest material, making them invisible. During incubation, the female removes the covering material, fully exposing all eggs. This shift in egg visibility makes the green‐backed tit a compelling model for studying the plasticity of egg recognition. Although there are no records of cuckoo parasitism in this species, it possesses a strong egg recognition capacity to reject 100% of nonmimetic eggs and an intermediate proportion of mimetic eggs (Yang et al. 2019). Such egg recognition capacity was stronger than its sibling species great tit ( *P. major* ), which has been recorded as a host of the common cuckoo ( *Cuculus canorus* ) (Liang et al. 2016; Liu and Yang 2026). Therefore, despite the current lack of natural parasitism records, the green‐backed tit provides an excellent case for testing hypotheses about the evolution of anti‐parasitic defenses, especially stage‐specific behavioral adaptations.

### 2.2 Field Procedure

**FIGURE 1:**
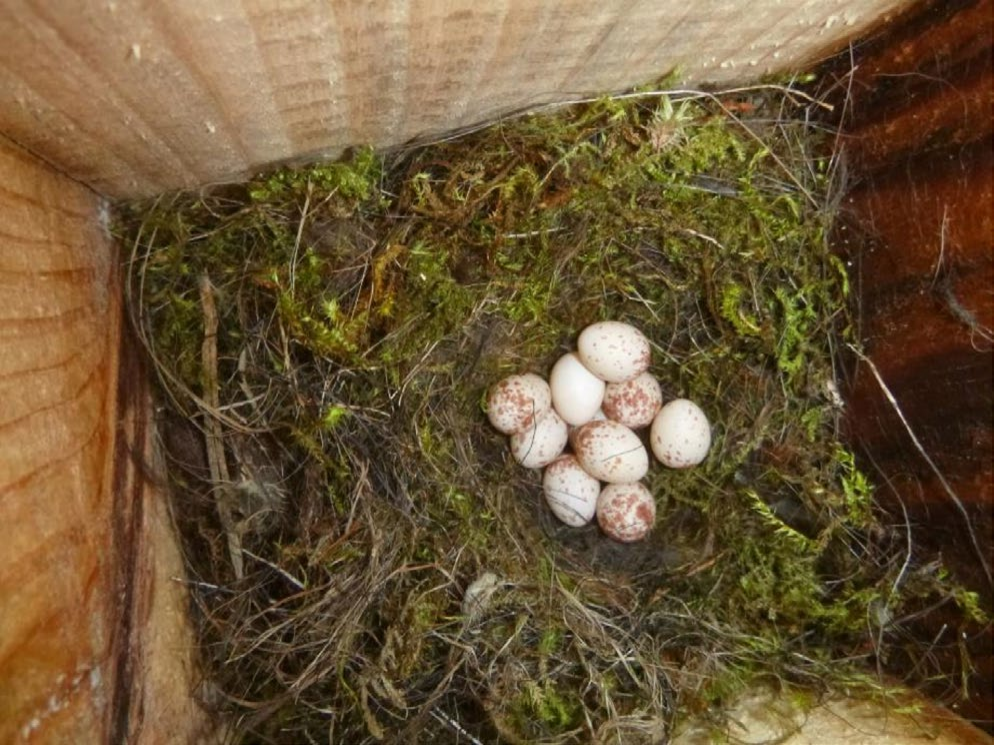
A case of using a munia (*immaculate white*) egg (the immaculate white egg) for parasitism experiment in a nest of the green‐backed tit.

During the breeding season, nest boxes were surveyed twice per week to confirm their occupied status by the hosts. When nest materials were detected, nest boxes were inspected every other day to confirm the hosts' egg‐laying time and clutch size. In the parasitism experiment, two treatments were arranged. In the first treatment, the parasitism experiment was conducted during the egg‐laying stage of each nest and monitored until the 6th day of incubation. This means the same individual was parasitized during laying and monitored through incubation, allowing us to evaluate within‐individual changes in response across different reproductive stages. In the second treatment, the parasitism experiment was performed only during the egg‐incubation stage (on the day after the clutches were completed) and monitored for 6 days. The results were then compared with those from the egg‐laying stage in the first treatment. This allows us to compare the response to parasitism occurring solely during incubation with the response during laying from treatment one at the population level. In other words, the first treatment provided a within‐individual, longitudinal comparison of responses across laying and incubation stages. The second treatment provided a population‐level, cross‐sectional comparison of responses when parasitism occurred during incubation versus laying. In both treatments, we used commercially purchased eggs from white‐rumped munia ( *Lonchura striata* ) rather than the commonly used model eggs to represent the parasite eggs because previous study has found that model eggs may be unable to detect the fine‐tuned recognition to parasite eggs in hosts when the parasitism risks change (Yang et al. 2020). Specifically, the hosts with lower rejection motivation to parasite eggs could not be detected by using model eggs because they were more difficult to reject than real eggs that would finally lead to compromised acceptance by the hosts (Yang et al. 2020). The munia eggs were immaculate white with a similar size to the host eggs (egg volume: 1285.54 ± 39.11 mm^3^ vs. 1309.96 ± 18.89 mm^3^, *n* = 15 in munias and tits, respectively), thus their phenotype represents nonmimetic parasite eggs to the host eggs (Figure 1). For the first treatment, a munia egg was inserted into a host nest during the egg‐laying stage to replace one host egg, and subsequently checked every 24 h until the 6th day of incubation. For the second treatment, the procedure was the same but manipulation was instead performed in the egg‐incubation stage and then checked every 24 h until the 6th day. Host responses were classified as rejection if the parasite eggs were ejected, buried, or deserted. By contrast, the parasite eggs were regarded as accepted when they were intact and continuously incubated by hosts. Each observed nest received only one treatment of either egg‐laying or egg‐incubation stage. A control group (*n* = 15) was subjected to the same experimental procedure but without parasite egg manipulation conducted to account for the disturbances of human handling. As a result, no nest desertion or other abnormal phenomena were found in the control group. Critically, all experimental procedures simulated the natural breeding conditions of green‐backed tits. During the laying stage, eggs (including the experimental egg) were covered with nest material and thus visually inaccessible. During incubation, eggs were left uncovered and fully exposed.

### 2.3 Statistical Analyses

Generalized linear mixed models (GLMMs) based on the Markov Chain Monte Carlo technique (MCMC) were utilized to assess the effects of experimental parasitism on host responses. Two models were established according to two aforementioned treatments. The host response (i.e., ejection or acceptance of parasite eggs) was included as the response variable while the nest identity was used as the random effect in both models. The fixed effects in the first model included the stage (i.e., egg‐laying vs. egg‐incubation stage of a nest), laid day of experiment (i.e., the number of days in egg‐laying stage when experiment was performed), and clutch size. In the second model the fixed effects were the treatment type, the laying date (laying date of the first egg as a representation of breeding time), and clutch size. For the delay time of egg recognition that was recorded as the number of days based on a daily basis, it was used as a response variable in the Cox proportional‐hazards models (Cox models) to investigate the difference between stages or trials above. The Cox models were appropriate to analyze such time data because both the latency and the occurrence of an event were taken into account (Stevens et al. 2008), thus allow us to compare the delay time of egg recognition by using the survival probabilities of parasite eggs in host nests. Kaplan–Meier survival curves were generated to illustrate such probabilities. The GLMMs, Cox models, and Kaplan–Meier survival curves were executed using the packages *MCMCglmm*, *survminer* and *survival*, respectively, in R (v.4.2.2) for Windows (https://www.r‐project.org/).

## 3 Results

**FIGURE 2:**
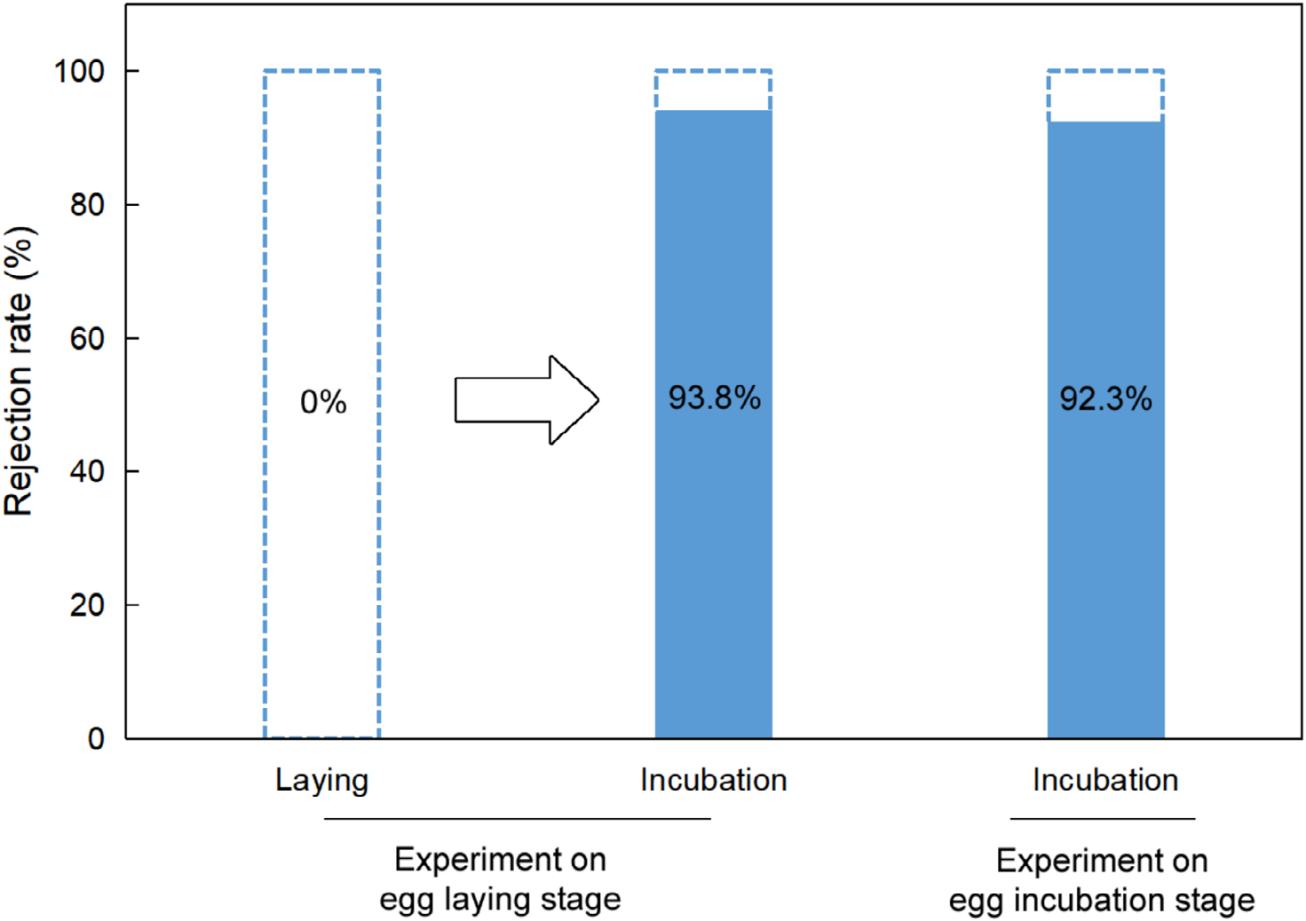
The results showing the rejection rates of foreign eggs from egg‐laying to egg‐incubation stages in egg‐laying trial (parasitism experiment on egg‐laying stage) and rejection rate of foreign eggs in egg‐incubation trial (parasitism experiment on egg‐incubation stage) by green‐backed tit.

**FIGURE 3:**
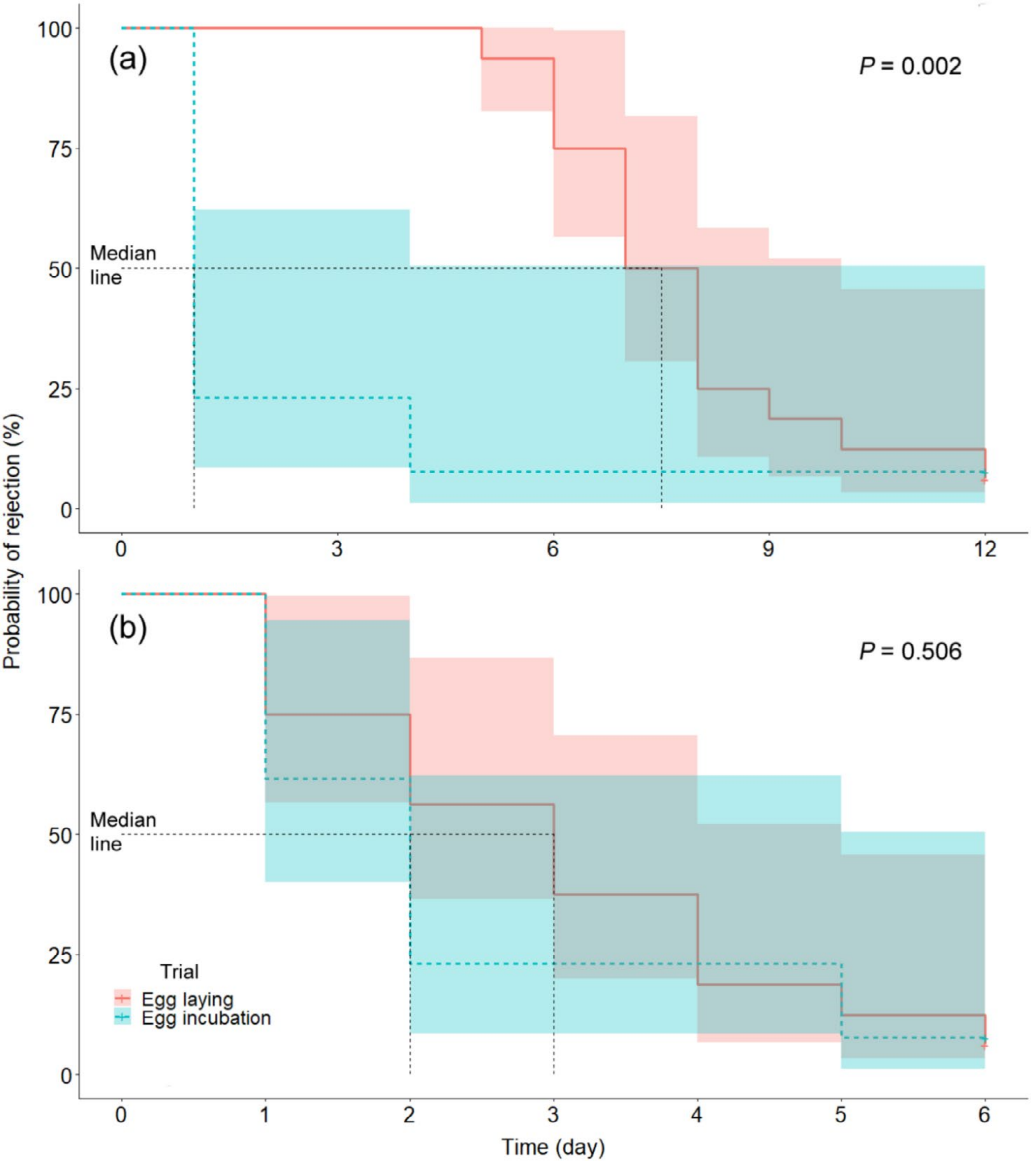
The Kaplan–Meier survival curves for rejection latency of the egg‐laying and egg‐incubation trials by green‐backed tits (mean ± 95% CI). (a) Rejection latency starts from the manipulation of parasitism experiment; (b) Rejection latency of (a) after excluding the days in laying stage. Egg‐laying and egg‐incubation trial The *p* values were calculated by Cox regression models.

According to the analyses, the host response of rejecting or accepting a parasite egg could be predicted by the breeding stages of a host individual, but neither the laid day of experiment nor clutch size (Table 1). In the first treatment when the experiment was performed during egg‐laying stage, the hosts accepted all parasite eggs before the clutches completed but rejected 93.8% of them during egg‐incubation stage (*n* = 16) (Figure 2). In the second treatment, 92.3% of parasite eggs (*n* = 13) were rejected, a rate which did not differ from that observed during the incubation stage in the first treatment (Table 2). Moreover, the laying date and clutch size did not predict the rejection rate either (Table 2). The survival probability of parasite eggs in host nests was significantly higher in the first treatment than in the second one (Figure 3) because the hosts did not react to the parasite eggs during the whole time of egg‐laying stage. However, if we excluded the delay time of egg‐laying stage from the first treatment, the survival probability of parasite eggs did not differ between the two treatments (Figure 3).

**TABLE 1:** The results of generalized linear mixed model using Markov chain Monte Carlo techniques comparing the egg rejection rates of green‐backed tits from egg‐laying to egg‐incubation stages in egg laying trial (parasitism experiment on egg‐laying stage).

	Posterior mean	Lower 95% CI	Upper 95% CI	*p*‐MCMC
Intercept	−0.78476	−1.26431	−0.18869	0.006**
Stage	0.93994	0.81395	1.05931	< 0.001***
Laid day of experiment	0.03632	−0.01145	0.08674	0.150^ns^
Clutch size	−0.03420	−0.10275	0.03233	0.332^ns^

*Note:* Random effect: nest identities; Stage: egg‐laying and egg‐incubation stages; Laid day of experiment: days of egg laying when experiment was performed; *p* ≥ 0.05^ns^; *p* < 0.05*; *p* < 0.01**; *p* < 0.001***.

**TABLE 2:** The results of generalized linear mixed model using Markov chain Monte Carlo techniques comparing the final egg rejection rates of green‐backed tits between egg‐laying and egg‐incubation trials.

	Posterior mean	Lower 95% CI	Upper 95% CI	*p*‐MCMC
Intercept	1.13833	0.52795	1.79293	< 0.001***
Treatment type	−0.15212	−0.44926	0.14296	0.306^ns^
Laying date	−0.02951	−0.07275	0.01587	0.190^ns^
Clutch size	0.02939	−0.04311	0.10074	0.426^ns^

*Note:* Random effect: nest identities; Treatment type: experiment on egg‐laying stage (the first treatment) and on egg‐incubation stage (the second treatment); Laying date: laying date of the first egg; *p* ≥ 0.05^ns^; *p* < 0.05*; *p* < 0.01**; *p* < 0.001***.

## 4 Discussion

This study elucidates that even though the green‐backed tits possess a strong capacity for egg recognition against parasite eggs, they did not express any rejection of parasite eggs during the egg‐laying stage, but instead rejected them during the egg‐incubation stage. In other words, for the green‐backed tits, the egg recognition or rejection was not triggered during the egg‐laying stage, but only after their clutches were completed. These results contradict the conclusions of previous studies that egg rejection was stronger in the egg‐laying than the egg‐incubation stage (Liu et al. 2021; Moksnes et al. 1991; Moskát et al. 2014; Wang et al. 2015), wherein Liu et al. (2021) found this effect even when the parasitism was relaxed. We propose that this biphasic pattern can possibly be explained by the egg visibility. As we have observed, the green‐backed tits would cover their eggs with nest materials (generally by moss) during the egg‐laying stage, making the eggs visually inaccessible. This concealment likely suppresses the host's ability to discriminate and reject foreign eggs through visual cues. Such egg‐covering behavior, also documented in other tits (Loukola et al. 2020; Saavedra and Amo 2019; Slagsvold and Wiebe 2021), is considered an adaptation against nest predation (Liu, Zhang, et al. 2023; Loukola et al. 2020; Saavedra and Amo 2019). Thus, during the laying stage, avoiding nest predation appears to take precedence over anti‐parasitic defense, even at the temporary cost of increased vulnerability to brood parasitism. During the incubation stage, the eggs remain uncovered and fully visible, allowing the expression of the innate egg‐recognition capacity. Together with the high cost of accepting a foreign egg at this stage (Davies 2000), this leads to the near‐universal rejection observed in our experiments. Therefore, the observed pattern does not imply an absence of recognition during laying, but rather its conditional suppression under a different selection pressure.

The evolution of egg‐recognition behavior may be driven by multiple selection pressures, such as defense against interspecific or intraspecific brood parasitism, or the removal of nonviable eggs. However, neither intraspecific parasitism nor egg‐viability filtering appears to be a primary driver in this species, as green‐backed tits do not reject conspecific eggs (Ye et al. 2022), nor do they remove nonviable eggs, as observed in previous works. Therefore, the most appropriate explanation for their well‐developed egg recognition is defense against interspecific brood parasites, which may have evolved under historical parasitism pressure and persists as a latent adaptation. Furthermore, our results imply that the green‐backed tits did not recognize the parasite eggs by imprinting on the first egg they laid. Recent theories hypothesized the hosts would recognize the parasite egg by imprinting their eggs (Liu, Wang, and Liang 2023; Ma and Liang 2021; Yang et al. 2022) or following a rule of discordance (Moskát et al. 2010; Yang et al. 2014). The former hypothesis indicates that the hosts would learn to recognize their egg phenotype by imprinting on the first egg they laid (Rothstein 1974, 1978; Wang et al. 2015; Yang et al. 2014) or the first clutch they laid (Lotem et al. 1995; Stokke et al. 2007) as a template to discriminate the parasite eggs. As the green‐backed tits did not recognize the parasite eggs during egg‐laying stage, imprinting on the first laid egg could be excluded. Except for learning by imprinting, the hosts were also assumed to use a nonlearning mechanism to spot the parasite eggs. The recognition by discordance refers to a mechanism that the hosts would reject the eggs of minority in their nests, regardless of host or parasite eggs (Moskát et al. 2010; Yang et al. 2014). However, it could not explain our results because previous study has excluded such recognition mechanism in this studied population of green‐backed tits (Zhao et al. 2024). Actually, none of the previous studies has found that the hosts would use discordance mechanism for egg recognition (Liu, Wang, and Liang 2023; Ma and Liang 2021; Yang et al. 2022), except for a study showing that the ashy‐throated parrotbills ( *Paradoxornis alphonsianus* ) used a combined mechanism of both template and discordance to reject parasite eggs (Yang et al. 2014). This study hypothesized that the discordance mechanism may be used by the male parrotbills because this hosts lay distinctly polymorphic eggs that the males were supposed to avoid rejecting their own egg by mistake when they paired with females of different egg phenotype (Liang et al. 2012; Yang et al. 2014).

Other variables, including the breeding time (represented by the egg laying date) did not predict egg recognition of the hosts. This indicates that the egg recognition capacity of green‐backed tits did not vary with time in a breeding season. According to previous theories, egg recognition was supposed to vary with time in a breeding season because of fluctuations in parasitism risks with the peak of cuckoo parasitism (Brooke et al. 1998; Liu et al. 2021; Moskát 2005; Zhang et al. 2021, 2022) or age/individual experience (Amundsen et al. 2002; Hauber et al. 2006; Lotem et al. 1992). Nevertheless, previous evidence that proved or disproved these theories was rather mixed. For example, great reed warblers (Brooke et al. 1998; Thorogood and Davies 2013) and rufous‐tailed scrub robins ( *Cercotrichas galactotes* ) (Álvarez 1996; Soler et al. 2012) show lower rejection later in the season or after cuckoos depart, whereas Daurian redstarts increase rejection rate following cuckoo arrival (Zhang et al. 2021). In contrast, species such as the isabelline shrike ( *Lanius isabellinus* ) (Zhou and Liang 2024) and European blackbirds ( *Turdus merula* ) (Grim et al. 2014) exhibit no seasonal variation in rejection behavior. Thus, the absence of seasonal plasticity in green‐backed tits suggests that their egg‐recognition behavior is not fine‐tuned to temporal changes in parasitism risk, but instead reflects a stable defensive trait.

We acknowledge that our experiment simulated parasitism only via egg addition, without cues of adult parasite (e.g., visual or sound signals), which in other systems can modulate host risk perception and rejection decisions (Tryjanowski et al. 2021). The observed biphasic pattern may represent a fixed behavioral program under the conditions of our study. Future studies integrating simulated parasite presence with egg‐addition experiments could test whether this strategy is further fine‐tuned by the host's immediate assessment of parasitic threat.

In summary, this study demonstrates that green‐backed tits exhibit a unique temporal pattern of egg rejection, characterized by complete acceptance of foreign eggs during the egg‐laying stage followed by near‐universal rejection during incubation. This behavior contrasts sharply with patterns observed in other host species, where rejection rates typically decline postlaying. We propose that this dichotomy reflects independent adaptations to distinct selective pressures: nest predation driving egg‐covering behavior during laying (suppressing rejection triggers) and brood parasitism favoring robust egg recognition during incubation. These findings challenge the assumption that egg rejection strategies are uniformly shaped by parasitism risk alone, highlighting the role of multifactorial selection in shaping host defenses.

## Author Contributions

**Ping Ye:** formal analysis (equal), investigation (equal), writing – original draft (equal). **Canchao Yang:** conceptualization (equal), formal analysis (equal), funding acquisition (equal), methodology (equal), resources (equal), supervision (equal), validation (equal), writing – original draft (equal), writing – review and editing (equal).

## Funding

Financial support has been provided by the Hainan Provincial Natural Science Foundation of China (No. 326JCQN0973) and National Natural Science Foundation of China (No. 32260127) to C.Y. and Doctoral Start‐up Funds of China West Normal University (No. 493002) to P.Y.

## Ethics Statement

The experiments reported here comply with the current laws of China. Fieldwork was carried out under permission from Kuankuoshui National Nature Reserve, P.R. China. Experimental procedures were in agreement with the Animal Research Ethics Committee of Hainan Provincial Education Centre for Ecology and Environment, Hainan Normal University (permit no. HNECEE2011‐001).

## Conflicts of Interest

The authors declare no conflicts of interest.

## Supporting information


**Data S1:** ece373404‐sup‐0001‐DataS1.xls.

## Data Availability

Data were provided as supporting information.

## References

[ece373404-bib-0001] Álvarez, F. 1996. “Model Cuckoo *cuculus canorus* Eggs Accepted by Rufous Bush Chats *Cercotrichas galactotes* During the Parasite's Absence From the Breeding Area.” Ibis 138: 340–342.

[ece373404-bib-0002] Amundsen, T. , P. T. Brobakken , A. Moksnes , and E. Røskaft . 2002. “Rejection of Common Cuckoo *Cuculus canorus* Eggs in Relation to Female Age in the Bluethroat *Luscinia svecica* .” Journal of Avian Biology 33, no. 4: 366–370. 10.1034/j.1600-048X.2002.02894.x.

[ece373404-bib-0003] Brooke, M. L. , N. B. Davies , and D. G. Noble . 1998. “Rapid Decline of Host Defences in Response to Reduced Cuckoo Parasitism: Behavioural Flexibility of Reed Warblers in a Changing World.” Proceedings of the Royal Society B: Biological Sciences 265, no. 1403: 1277–1282. 10.1098/rspb.1998.0430.

[ece373404-bib-0004] Davies, N. B. 2000. Cuckoos, Cowbirds and Other Cheats. T & A Poyser.

[ece373404-bib-0005] Davies, N. B. 2011. “Cuckoo Adaptations: Trickery and Tuning.” Journal of Zoology 284, no. 1: 1–14. 10.1111/j.1469-7998.2011.00810.x.

[ece373404-bib-0006] Davies, N. B. , and M. d. L. Brooke . 1989. “An Experimental Study of Co‐Evolution Between the Cuckoo Cuculus Canorus and Its Hosts. I. Host Egg Discrimination.” Journal of Animal Ecology 58, no. 1: 207–224. 10.2307/4996.

[ece373404-bib-0007] Grim, T. , P. Samaš , and M. E. Hauber . 2014. “The Repeatability of Avian Egg Ejection Behaviors Across Different Temporal Scales, Breeding Stages, Female Ages and Experiences.” Behavioral Ecology and Sociobiology 68, no. 5: 749–759. 10.1007/s00265-014-1688-9.

[ece373404-bib-0008] Grim, T. , P. Samaš , C. Moskát , et al. 2011. “Constraints on Host Choice: Why Do Parasitic Birds Rarely Exploit Some Common Potential Hosts?” Journal of Animal Ecology 80, no. 3: 508–518. 10.1111/j.1365-2656.2010.01798.x. 21244420

[ece373404-bib-0010] Hauber, M. E. , C. Moskát , and M. Bán . 2006. “Experimental Shift in Hosts' Acceptance Threshold of Inaccurate‐Mimic Brood Parasite Eggs.” Biology Letters 2, no. 2: 177–180. 10.1098/rsbl.2005.0438. 17148357 PMC1618923

[ece373404-bib-0011] Liang, W. , A. P. Møller , B. G. Stokke , et al. 2016. “Geographic Variation in Egg Ejection Rate by Great Tits Across 2 Continents.” Behavioral Ecology 27, no. 5: 1405–1412. 10.1093/beheco/arw061.

[ece373404-bib-0012] Liang, W. , C. Yang , A. Antonov , et al. 2012. “Sex Roles in Egg Recognition and Egg Polymorphism in Avian Brood Parasitism.” Behavioral Ecology 23, no. 2: 397–402. 10.1093/beheco/arr203.

[ece373404-bib-0013] Liu, C. , P. Ye , Y. Cai , R. Quan , and C. Yang . 2021. “Persistent Fine‐Tuning of Egg Rejection Based on Parasitic Timing in a Cuckoo Host Even After Relaxation of Parasitism Pressure.” Behavioural Processes 193: 104532. 10.1016/j.beproc.2021.104532. 34648869

[ece373404-bib-0014] Liu, J. , L. Wang , and W. Liang . 2023. “Egg Rejection and Egg Recognition Mechanism in a Chinese Azure‐Winged Magpie ( *Cyanopica cyanus* ) Population.” Avian Research 14: 100112. 10.1016/j.avrs.2023.100112.

[ece373404-bib-0015] Liu, J. , F. Zhang , X. Zhang , and W. Liang . 2023. “Egg Covering by Cavity‐Nesting Birds: An Experimental Test of the Usurpation Hypothesis.” Behavioral Ecology and Sociobiology 77, no. 9: 103. 10.1007/s00265-023-03377-9.

[ece373404-bib-0016] Liu, T. , and C. Yang . 2026. “Host Exploitation by Cuckoos in China: A Review and Real‐Time Tracking Program for Parasitism Records.” Integrative Zoology 21: 218–230. 10.1111/1749-4877.13009. 40673901 PMC12971627

[ece373404-bib-0017] Lotem, A. , H. Nakamura , and A. Zahavi . 1992. “Rejection of Cuckoo Eggs in Relation to Host Age: A Possible Evolutionary Equilibrium.” Behavioral Ecology 3, no. 2: 128–132. 10.1093/beheco/3.2.128.

[ece373404-bib-0018] Lotem, A. , H. Nakamura , and A. Zahavi . 1995. “Constraints on Egg Discrimination and Cuckoo–Host Co‐Evolution.” Animal Behaviour 49, no. 5: 1185–1209. 10.1006/anbe.1995.0152.

[ece373404-bib-0019] Loukola, O. J. , P. Adamik , F. Adriaensen , et al. 2020. “The Roles of Temperature, Nest Predators and Information Parasites for Geographical Variation in Egg Covering Behaviour of Tits (Paridae).” Journal of Biogeography 47, no. 7: 1482–1493. 10.1111/jbi.13830.

[ece373404-bib-0020] Ma, L. , and W. Liang . 2021. “Egg Rejection and Egg Recognition Mechanisms in Oriental Reed Warblers.” Avian Research 12: 47. 10.1186/s40657-021-00283-4.

[ece373404-bib-0021] Ma, L. , W. Liu , P. Pan , J. Hou , and W. Liang . 2024. “Oriental Reed Warblers Retain Strong Egg Recognition Abilities During the Nestling Stage.” Ecology and Evolution 14, no. 2: e11063. 10.1002/ece3.11063. 38380067 PMC10877555

[ece373404-bib-0022] Moksnes, A. , E. Røskaft , A. T. Braa , L. Korsnes , H. M. Lampe , and H. C. Pedersen . 1991. “Behavioural Responses of Potential Hosts Towards Artificial Cuckoo Eggs and Dummies.” Behaviour 116, no. 1: 64–89. 10.1163/156853990X00365.

[ece373404-bib-0023] Moskát, C. 2005. “Nest Defence and Egg Rejection in Great Reed Warblers Over the Breeding Cycle: Are They Synchronised With the Risk of Brood Parasitism?” Annales Zoologici Fennici 42, no. 6: 579–586.

[ece373404-bib-0024] Moskát, C. , M. Bán , T. Székely , et al. 2010. “Discordancy or Template‐Based Recognition? Dissecting the Cognitive Basis of the Rejection of Foreign Eggs in Hosts of Avian Brood Parasites.” Journal of Experimental Biology 213, no. 11: 1976–1983. 10.1242/jeb.040394. 20472785

[ece373404-bib-0025] Moskát, C. , M. E. Hauber , Z. Elek , et al. 2014. “Foreign Egg Retention by Avian Hosts in Repeated Brood Parasitism: Why Do Rejecters Accept?” Behavioral Ecology and Sociobiology 68, no. 3: 403–413. 10.1007/s00265-013-1654-y.

[ece373404-bib-0026] Payne, R. B. 2005. The Cuckoos. Oxford University Press.

[ece373404-bib-0027] Rothstein, S. I. 1974. “Mechanisms of Avian Egg Recognition: Possible Learned and Innate Factors.” Auk 91, no. 4: 796–807. 10.2307/4084731.

[ece373404-bib-0028] Rothstein, S. I. 1978. “Mechanisms of Avian Egg‐Recognition: Additional Evidence for Learned Components.” Animal Behaviour 26: 671–677. 10.1016/0003-3472(78)90133-1.

[ece373404-bib-0029] Saavedra, I. , and L. Amo . 2019. “Egg Concealment Is an Antipredatory Strategy in a Cavity‐Nesting Bird.” Ethology 125, no. 11: 785–790. 10.1111/eth.12932.

[ece373404-bib-0031] Slagsvold, T. , and K. L. Wiebe . 2021. “Egg Covering in Cavity Nesting Birds May Prevent Nest Usurpation by Other Species.” Behavioral Ecology and Sociobiology 75, no. 8: 116. 10.1007/s00265-021-03045-w. 34759442 PMC8570351

[ece373404-bib-0032] Soler, M. 2014. “Long‐Term Coevolution Between Avian Brood Parasites and Their Hosts.” Biological Reviews 89, no. 3: 688–704. 10.1111/brv.12075. 24330159

[ece373404-bib-0033] Soler, M. , M. Martín‐Vivaldi , and J. Fernández‐Morante . 2012. “Conditional Response by Hosts to Parasitic Eggs: The Extreme Case of the Rufous‐Tailed Scrub Robin.” Animal Behaviour 84, no. 2: 421–426. 10.1016/j.anbehav.2012.05.016.

[ece373404-bib-0034] Soler, M. , T. Pérez‐Contreras , and J. J. Soler . 2020. “Great Spotted Cuckoos Show Dynamic Patterns of Host Selection During the Breeding Season. The Importance of Laying Stage and Parasitism Status of Magpie Nests.” Behavioral Ecology 31, no. 2: 467–474. 10.1093/beheco/arz208.

[ece373404-bib-0035] Stevens, M. , C. J. Hardman , and C. L. Stubbins . 2008. “Conspicuousness, Not Eye Mimicry, Makes “Eyespots” Effective Antipredator Signals.” Behavioral Ecology 19, no. 3: 525–531. 10.1093/beheco/arm162.

[ece373404-bib-0036] Stokke, B. G. , E. Roskaft , and A. Moksnes . 2016. “Disappearance of Eggs From Nonparasitized Nests of Brood Parasite Hosts: The Evolutionary Equilibrium Hypothesis Revisited.” Biological Journal of the Linnean Society 118, no. 2: 215–225. 10.1111/bij.12733.

[ece373404-bib-0037] Stokke, B. G. , F. Takasu , A. Moksnes , and E. Røskaft . 2007. “The Importance of Clutch Characteristics and Learning for Antiparasite Adaptations in Hosts of Avian Brood Parasites.” Evolution 61, no. 9: 2212–2228. 10.1111/j.1558-5646.2007.00176.x. 17767591

[ece373404-bib-0038] Thorogood, R. , and N. B. Davies . 2013. “Reed Warbler Hosts Fine‐Tune Their Defenses to Track Three Decades of Cuckoo Decline.” Evolution 67, no. 12: 3545–3555. 10.1111/evo.12213. 24299407 PMC4209118

[ece373404-bib-0039] Tryjanowski, P. , A. Golawski , M. Janowski , and T. H. Sparks . 2021. “Does Experimentally Simulated Presence of a Common Cuckoo( *Cuculus canorus* ) Affect Egg Rejection and Breeding Success in the Red‐Backed Shrike ( *Lanius collurio* )?” Acta Ethologica 24, no. 2: 87–94. 10.1007/s10211-021-00362-1.

[ece373404-bib-0040] Underwood, T. J. , and S. G. Sealy . 2006. “Parameters of Brown‐Headed Cowbird *Molothrus ater* Egg Discrimination in Warbling Vireos *Vireo gilvus* .” Journal of Avian Biology 37, no. 5: 457–466. 10.1111/j.2006.0908-8857.03583.x.

[ece373404-bib-0041] Visser, M. E. , and C. M. Lessells . 2001. “The Costs of Egg Production and Incubation in Great Tits ( *Parus major* ).” Proceedings of the Royal Society B: Biological Sciences 268, no. 1473: 1271–1277. 10.1098/rspb.2001.1661. PMC1088737 11410154

[ece373404-bib-0042] Wang, L. , C. Yang , A. P. Møller , W. Liang , and X. Lu . 2015. “Multiple Mechanisms of Egg Recognition in a Cuckoo Host.” Behavioral Ecology and Sociobiology 69, no. 11: 1761–1767. 10.1007/s00265-015-1988-8.

[ece373404-bib-0043] Yang, C. , X. Chen , L. Wang , and W. Liang . 2022. “Defensive Adaptations to Cuckoo Parasitism in the Black‐Browed Reed Warbler ( *Acrocephalus bistrigiceps* ): Recognition and Mechanism.” Animal Cognition 25, no. 5: 1299–1306. 10.1007/s10071-022-01613-9. 35320446

[ece373404-bib-0044] Yang, C. , W. Liang , and A. P. Møller . 2019. “Egg Retrieval Versus Egg Rejection in Cuckoo Hosts.” Philosophical Transactions of the Royal Society, B: Biological Sciences 374: 20180200. 10.1098/rstb.2018.0200. PMC6388044 30967079

[ece373404-bib-0045] Yang, C. , A. P. Møller , E. Røskaft , A. Moksnes , W. Liang , and B. G. Stokke . 2014. “Reject the Odd Egg: Egg Recognition Mechanisms in Parrotbills.” Behavioral Ecology 25, no. 6: 1320–1324. 10.1093/beheco/aru124.

[ece373404-bib-0046] Yang, C. , L. Wang , S. J. Cheng , Y. C. Hsu , A. P. Moller , and W. Liang . 2020. “Model Eggs Fail to Detect Egg Recognition in Host Populations After Brood Parasitism Is Relaxed.” Frontiers in Zoology 17: 14. 10.1186/s12983-020-00362-0. 32426018 PMC7216403

[ece373404-bib-0047] Yang, C. , P. Ye , N. Wu , X. Yao , and W. Liang . 2023. “Revealing the Roles of Egg Darkness and Nest Similarity for a Cryptic Parasite Egg Versus Host's Cognition: An Alternate Coevolutionary Trajectory.” Proceedings of the Royal Society B: Biological Sciences 290, no. 1998: 20230103. 10.1098/rspb.2023.0103. PMC10154918 37132235

[ece373404-bib-0048] Ye, P. , Y. Cai , J. Bi , X. Yao , G. Li , and C. Yang . 2022. “Parasite Egg Recognition Based on Multicomponent Cues by Green‐Backed Tits ( *Parus monticolus* ).” Avian Biology Research 15, no. 1: 53–57. 10.1177/17581559211066092.

[ece373404-bib-0049] Ye, P. , X. Yao , J. Bi , G. Li , W. Liang , and C. Yang . 2021. “Breeding Biology of the Green‐Backed Tit ( *Parus monticolus* ) in Southwest China.” Avian Research 12: 60. 10.1186/s40657-021-00296-z.

[ece373404-bib-0050] Zhang, J. , A. P. Møller , D. Yan , J. Li , and W. Deng . 2021. “Egg Rejection Changes With Seasonal Variation in Risk of Cuckoo Parasitism in Daurian Redstarts, *Phoenicurus auroreus* .” Animal Behaviour 175: 193–200. 10.1016/j.anbehav.2021.03.007.

[ece373404-bib-0051] Zhang, J. , P. Santema , J. Li , W. E. Feeney , W. Deng , and B. Kempenaers . 2022. “The Mere Presence of Cuckoos in Breeding Area Alters Egg‐Ejection Decisions in Daurian Redstarts.” Behavioral Ecology 33, no. 6: 1153–1160. 10.1093/beheco/arac084.

[ece373404-bib-0052] Zhao, X. , P. Ye , H. Zhou , and C. Yang . 2024. “Effect of Parasite Egg Size and Quantity Contrast of Parasite‐Host Eggs on Recognition and Rejection Mode of Green‐Backed Tits.” Avian Research 15: 100216. 10.1016/j.avrs.2024.100216.

[ece373404-bib-0053] Zhou, B. , and W. Liang . 2024. “Seasonal Increase in Nest Defense, but Not Egg Rejection, in a Cuckoo Host.” Avian Research 15: 100154. 10.1016/j.avrs.2023.100154.

